# The Use of Methylene Blue in Conjunction With Hydroxocobalamin and Multiple Pressors to Treat Severe Vasoplegia in a Patient Due to Calcium Channel Blocker Toxicity: A Case Report

**DOI:** 10.7759/cureus.53778

**Published:** 2024-02-07

**Authors:** Aaron Hacker, Dylan S Irvine, Michael Gross, Imani Thornton, Diego Marin

**Affiliations:** 1 Anesthesiology, Health Corporation of America (HCA) Florida Westside Hospital, Plantation, USA; 2 Osteopathic Medicine, Nova Southeastern University, Dr. Kiran C. Patel College of Osteopathic Medicine, Fort Lauderdale, USA; 3 Critical Care, Health Corporation of America (HCA) Florida Westside Hospital, Plantation, USA

**Keywords:** critical care medicine, methylene blue, calcium channel blockers, anesthesiology, hydroxocobalamin, vasoplegia

## Abstract

Vasoplegia, the demonstration of persistently low systemic vascular resistance (SVR) and resistant hypotension in the presence of a normal cardiac index despite aggressive resuscitation attempts, is a serious clinical diagnosis that requires prompt treatment to prevent patient morbidity and mortality. Currently, treatment of vasoplegia involves treatment with vasopressors such as vasopressin, norepinephrine, and hydroxocobalamin. However, some evidence suggests that in addition to this treatment regimen, the addition of methylene blue may result in a reduction in overall norepinephrine equivalent vasopressor requirements, increased mean arterial pressure, and an improved clinical course. Here, we report the case of a 64-year-old male patient who presented to the ED after being found unresponsive and covered in emesis at home. The patient’s presentation was complicated by worsening dyspnea, hypotension, and hemodynamic instability, requiring intubation and admission to the ICU for management of undifferentiated shock of unclear etiology and acute respiratory failure. Urine studies were consistent with a diagnosis of vasoplegia due to dihydropyridine calcium channel blocker toxicity, which was confirmed by pill counting of his home medications in the setting of recent paranoia and depression. The patient was treated aggressively with vasopressors, including vasopressin, phenylephrine, and epinephrine, as well as a combination of hydroxocobalamin and methylene blue. He was also started on a calcium and insulin drip. Upon initiation of non-catecholamine agents for vasoplegia, his clinical course quickly improved, and he was weaned from all vasopressors. He regained hemodynamic stability, was successfully extubated, evaluated by psychiatry, and discharged from the hospital in a stable condition on day 15 with the continuation of outpatient psychiatric services.

## Introduction

Vasoplegia is characterized by persistently low systemic vascular resistance (SVR) following failed initial hypotensive management, including fluid bolus, and subsequent catecholamine escalation [1]. Vasoplegia commonly presents with vital signs reflecting a normal or high cardiac index, pulmonary capillary wedge pressure below 12 mmHg, and SVR below 900 dynnes/s/cm^5 [1]. Vasoplegia can also be catecholamine resistant, resulting in profound uncontrolled vasodilation and hypotension despite aggressive catecholamine escalation along with resuscitation attempts [2]. The etiologies of vasoplegia include sepsis, cardiovascular and cardiothoracic surgeries, including but not limited to cardiopulmonary bypass, medication overdoses, blood transfusions, organ transplantation, trauma, and sepsis [1,3-4]. The pathophysiology of vasoplegia is multifactorial but may include inducible nitric oxide synthase (iNOS) release as well as inflammatory cytokines serving as major contributors to inappropriate vasodilation [5-7]. Additionally, potassium-sensitive adenosine triphosphate (K-ATP) channels distributed in the vasculature regulate arterial tone and prevent calcium entry via depolarization of membrane potential, thus preventing vasoconstriction [5]. Clinical manifestations of vasoplegia include hypotension refractory to vasopressors, hyperglycemia, and altered mental status [1]. The diagnostic approach is primarily clinical, as other potential causes of hypotension must be ruled out. In the absence of absolute contraindications and given the appropriate clinical picture, management of refractory vasoplegia typically includes empiric treatment with catecholamine vasopressors such as norepinephrine, epinephrine, dopamine, and phenylephrine [1,5]. However, evidence suggests that administration of combination therapy with drugs with different mechanisms of action, such as the addition of non-catecholamine agents (e.g., vasopressin, angiotensin II, methylene blue, and hydroxocobalamin), results in greater increases in MAP and a reduction in overall norepinephrine equivalent vasopressor requirements [1,5].

## Case presentation

We report the case of a 64-year-old male who presented to the ED after being found by his spouse unresponsive and covered in emesis at home. The patient’s presentation was complicated by worsening dyspnea and hemodynamic instability, requiring intubation and vasopressor initiation. Physical examination was significant for dry mucous membranes with non-distended neck veins without bruits. A cardiac examination revealed tachycardia with a regular rhythm and no murmurs. Pulmonary auscultation demonstrated crackles and decreased breath sounds at bilateral lung bases. Abdominal and extremity examinations were benign, with no swelling, edema, or tenderness to palpation. Past medical history was significant for hypertension, dyslipidemia, depression, nephrolithiasis, and benign prostatic hypertension. His past surgical history was significant for lithotripsy two weeks prior to his current presentation. The patient’s home medications included amlodipine, atorvastatin, clonazepam, mirtazapine, and tamsulosin.

In the ED, CT scans of the head, chest, abdomen, and pelvis were within normal limits and ruled out acute strokes or other acute processes. An EKG showed sinus tachycardia, and an echocardiogram revealed an estimated ejection fraction of 50% with mild hypokinesis of the left ventricle but normal left and right ventricular size. These examinations ruled out cardiogenic shock, tamponade, and significant valvulopathy. Further, CT angiography ruled out pulmonary embolism. Initial laboratory workup from the ED revealed leukocytosis, as shown in Table 1. Urinalysis was suggestive of a UTI as evidenced by cloudy yellow urine, positive leukocyte esterase, and high urine WBC, as shown in Table 2. A urine toxicology screen was negative. The patient was started on empiric antibiotics consisting of vancomycin and piperacillin/tazobactam and was admitted to the ICU for the management of refractory shock and acute hypoxic respiratory failure.

**Table 1:** CMP and CBC results taken on hospital day 1 (in the ED) and on hospital day 2 (day 1 in the ICU) BUN: blood urea nitrogen; EST GFR: estimated glomerular filtration rate; AST: aspartate aminotransferase; ALT: alanine aminotransferase; WBC: white blood cells; RBC: red blood cells; HGB: hemoglobin; HCT: hematocrit; MCV: mean corpuscular volume; MCHC: mean corpuscular hemoglobin concentration; RDW: red cell distribution width; PLT: platelet; MPV: mean platelet volume; L: low; H: high; HC: critical high; CMP: comprehensive metabolic panel; ED: emergency department; ICU: intensive care unit

Lab value (units)	Hospital day 1 (in the ED)	Hospital day 2 (day 1 in the ICU)	Reference range
CMP			
Sodium (mmol/L)	133 L	136	135-145
Potassium (mmol/L)	3.5	3.0 L	3.5-5.2
Chloride (mmol/L)	101	102	95-110
Carbon dioxide (mmol/L)	12 L	10 L	19-34
BUN (mg/dL)	20	20	6-33
Creatinine (mg/dL)	1.60 H	1.66 H	0.43-1.13
EST GFR (non-African American) (ml/min/L)	44	42	>60
Lactic acid (mmol/L)	5.9 CH	14.3 CH	0.4-2.0
Total bilirubin (mg/dL)	0.6	1.6 H	0.1-1.2
AST (units/L)	56	76	10-40
ALT (units/L)	43	56	10-60
Total alk phosphatase (units/L)	43	40	20-130
Creatinine kinase (units/L)	1265 H	1468 H	39-308
CBC			
WBC (cells per 10^3/uL)	16.6 H	13.3 H	4.0-10.5
RBC (cells per 10^6/uL)	3.80 L	4.56 L	4.63-6.08
HGB (g/dL)	10.7 L	11.8 L	13.7-17.5
HCT (%)	33.9 L	36.6	40.1-51.0
MCV (fl)	89.2	86.9	79.0-92.2
MCHC (g/dL)	31.6 L	32.2 L	32.3-36.5
RDW (%)	12.1	12.4	11.6-14.4
PLT count (cells per 10^3/uL)	193	204	150-400
MPV (fl)	10.1	9.6	9.4-12.4
Absolute neutrophils (cells per 10^3/uL)	12.51 H	9.56 H	1.56-6.13
Segmented neutrophils (%)	84.2 H	87.4 H	34.0-67.9
Lymphocytes (%)	7.0 L	0.68L	21.0-53.1
Monocytes (%)	0.94	5.1	5.3-12.2
Eosinophils (cells per 10^3/uL)	0.02 L	0.0	0.04-0.54

**Table 2:** Urinalysis results taken on hospital day 1 (in the ED) and on hospital day 2 (day 1 in the ICU) RBC: red blood cells; WBC: white blood cells; HPF: high power field; LPF: low power field; H: high; ED: emergency department, ICU: intensive care unit

Lab value (units)	Hospital day 1 (in the ED)	Hospital day 2 (day 1 in the ICU)	Reference range
Urine color	Yellow	Red H	Yellow
Urine appearance	Cloudy H	Turbid H	Clear
Urine pH	6.0	5.0	5.0-9.0
Urine specific gravity	1.040 H	1.059	1.005-1.035
Urine protein (mg/dL)	100 H	Negative	Negative
Urine ketones (mg/dL)	Negative	Negative	Negative
Urine blood	Small H	Large H	Negative
Urine nitrite	Negative	Negative	Negative
Urine leukocyte esterase	Small H	Negative	Negative
Urine RBC (cells per HPF)	21-50 H	21-50 H	0-5
Urine WBC (cells per HPF)	51-100 H	21-50 H	0-5
Urine mucus (cells per LPF)	Few H	Few H	None
Urine glucose (mg/dL)	Negative	50 H	Negative

Upon presentation to the ICU, the patient remained hypotensive and tachycardic, with a blood pressure (BP) of 78/38 mmHg and a heart rate of 104 beats per minute (bpm). The patient was initiated on vasopressor support, including norepinephrine, vasopressin, phenylephrine, and epinephrine. Despite traditional therapies and bolus-guided fluid resuscitation, mean arterial pressure (MAP) remained in the 40s to 50s, as measured with an Edwards hemisphere to monitor noninvasive BP, MAP, cardiac output (CO), and stroke volume (SV). The patient was mechanically ventilated and sedated with dexmedetomidine, propofol, and fentanyl infusions with Richmond Agitation Sedation Scale goals of -2, with oxygen saturations of 97% on a 40% fraction of inspired oxygen. He continued to remain in a refractory shock state despite aggressive management, which included a bicarbonate drip for his metabolic acidosis and guided fluid resuscitation.

A repeat urine assessment was conducted and is demonstrated in Table 2. Through further evaluation and a thorough history obtained from the patient’s family, a clinical diagnosis of vasoplegia due to calcium channel blocker (CCB) toxicity was suspected. Additional toxicology screening to include CCB overdose was ordered, and a home medication review including pill counting by the family was recommended. Treatment with 135 mg of methylene blue (2 mg/kg) and 5 g of hydroxocobalamin was initiated as infusions over a period of 10 minutes for the management of this undifferentiated, refractory vasoplegic shock. The administration of these agents correlated with a significant and timely improvement in our patient’s hemodynamics and decreased his vasopressor requirements, with MAPs able to be maintained above 65. The patient’s vitals, including heart rate, BP, MAP, CI, and SV, one hour before, just prior to, and one hour after the administration of methylene blue and hydroxocobalamin, can be seen in Table 3.

**Table 3:** Patient’s HR, BP, MAP, CI, and SV one hour before, 10 minutes before, and one hour after the administration of methylene blue and hydroxocobalamin HR: heart rate; BP: blood pressure; MAP: mean arterial pressure; CI: cardiac index; SV: stroke volume

Time	HR (beats per minute)	BP (mmHg)	MAP	CI (liter/minute/square meter)	SV (mL)
1 hour prior to administration	70	88/46	60	2.1	55
10 minutes prior to administration	76	90/50	64	2.3	60
1 hour after administration	81	109/58	80	2.5	65

The patient’s family brought in all of his medication bottles in the setting of recent-onset acute delirium, paranoia, and depression for pill counting verification. The possibility and likelihood of medication overdose were ascertained by the patient’s family, and it was determined that the patient ingested approximately a three-month supply of amlodipine within one day. The patient’s amlodipine screen confirmed this diagnosis of CCB toxicity, as shown in Table 4.

**Table 4:** Results of amlodipine screen, which was collected on hospital day 3 (day 2 in the ICU) ICU: intensive care unit

	Hospital day 3 (day 2 in the ICU)
Amlodipine screen	190 ng/ml (therapeutic serum range: 2-25 ng/ml)

The patient was started on a calcium and insulin infusion, a glucagon drip along with maintenance fluid, and periodic glucose checks. His clinical course quickly improved further, and he was weaned from all vasopressors and successfully extubated. The patient was evaluated by psychiatry and diagnosed with delirium, mood disorder, and paranoia. He was prescribed quetiapine 200 mg daily. The patient was discharged from the intensive care unit in stable condition on hospital day 11. The patient was discharged home with the continuation of outpatient psychiatric evaluation and treatment on hospital day 15.

## Discussion

Vasoplegia results in what is often termed vasodilatory or distributive shock and is characterized by decreased SVR, resulting in profound hypotension with end-organ hypoperfusion despite a normal or increased cardiac index [1]. There is little to no response of the arterioles to the catecholamine surge that results from the hypoperfusion, and vasopressors can similarly have a diminished response [1]. Vasoplegia is a known complication of sepsis and cardiovascular surgeries and is often seen in critical care settings [3-4]. Amlodipine, an oral dihydropyridine CCB, preferentially inhibits peripheral vascular and coronary calcium influx via slow channels, resulting clinically in decreased vascular tone [4,8]. Toxicity can occur with the unintentional or intentional intake of higher than prescribed doses and is a potential etiology of vasoplegia in patients. Other etiologies and risk factors for developing vasoplegia include blood transfusions, cardiopulmonary bypass, organ transplantation, trauma, sepsis, and the use of specific medications such as angiotensin-converting enzyme inhibitors, angiotensin-II antagonists, heparin, amiodarone, aprotinin, and protamine [1,9-10].

The pathophysiology of vasoplegia is multifactorial. Proposed mechanisms include inducible iNOS release, in this case, triggered by CCB toxicity, as well as inflammatory cytokines serving as major contributors to inappropriate vasodilation. Studies have confirmed in vivo and in vitro CCB-related increases in iNOS, NO production, iNOS activity, and iNOS protein expression [1]. iNOS produces nitric oxide, which increases vascular levels of cyclic guanosine monophosphate (cGMP), resulting in smooth muscle relaxation and subsequent vasodilation [1,11]. Additionally, K-ATP channels distributed in the vasculature regulate arterial tone and prevent calcium entry via depolarization of membrane potential, thus preventing vasoconstriction [1]. NO activates K-ATP, resulting in prolonged refractory states of calcium channels and thus further increasing vasoplegia [1]. Prolonged shock states cause a relative decrease in arginine-vasopressin and its activation. Vasopressin typically mediates vasoconstriction via vasopressin 1 and oxytocin receptors by way of increased intracellular calcium [1,12]. Vasopressin also modulates K-ATP, resulting in cell membrane hyperpolarization, typically blunting the NO-induced increased cGMP [1,12]. A subsidiary pathway mediator for vasoplegia is hydrogen sulfide (H2S), a byproduct of vitamin B6-dependent homocysteine metabolism [11]. In high-inflammatory states, H2S directly activates K-ATP channels, thus further reducing vascular tone [1,11].

Clinical manifestations of vasoplegia include hypotension refractory to vasopressors, hyperglycemia, and altered mental status [1-4]. The diagnostic approach is primarily clinical, as other potential causes of hypotension must be ruled out [1]. A clinical picture of significant arterial diastolic hypotension, normal or high CO or cardiac index, low SVR, and an increasing need for intravenous volume resuscitation and vasopressors should prompt consideration for vasoplegia as part of the working differential.

In the absence of absolute contraindications and given the appropriate clinical picture, management of refractory non-surgical vasoplegia such as the one presented here typically includes empiric treatment with catecholamine vasopressors such as norepinephrine, epinephrine, dopamine, and phenylephrine [1,4]. Norepinephrine is the best studied and is the first-line treatment agent. However, evidence suggests that administration of combination therapy of multiple catecholamines with different medications of action, such as the addition of non-catecholamine agents (e.g., methylene blue, hydroxocobalamin), results in greater increases in MAP and a reduction in overall norepinephrine equivalent vasopressor requirements [1,4-5,8-9,11-12]. Methylene blue reduces NO production via inhibition of iNOS, a rate-limiting step in NO production, as well as inhibition of guanylate cyclase [1]. Methylene blue may be contraindicated in patients with severe renal insufficiency or patients taking serotonergic medications due to its inhibition of monoamine oxidase (MOA-A) [1]. Hydroxocobalamin primarily induces an arterial BP response by direct inhibition of NO and iNOS, as well as modification of the endothelial-bound innate H2S groups [1]. Vasopressin also modulates K-ATP, resulting in cell membrane hyperpolarization, typically blunting the NO-induced increased cGMP [1]. The results of our case presentation support claims of a rapid increase in MAP and a reduction in the overall norepinephrine equivalent vasopressor requirements in severe refractory vasoplegia treated with methylene blue in conjunction with hydroxocobalamin and catecholamine vasopressors [1,4,12]. However, future studies should aim to test the efficacy of hydroxocobalamin alone versus hydroxocobalamin and methylene blue, in conjunction with catecholamine vasopressors, to treat refractory vasoplegia in a large patient sample. By reducing the total amount of catecholamines patients receive, there may be a reduction in the likelihood of cardiac toxicity or acute kidney injury, often associated with the inflammatory response, sepsis, or cardiopulmonary bypass. Methylene blue and hydroxocobalamin administration in conjunction with norepinephrine and vasopressin appears to be a safe and effective strategy for the management of patients in vasoplegic shock in the critical care setting.

## Conclusions

This case report describes the presentation of severe vasoplegic shock following the ingestion of approximately a three-month supply of amlodipine in a 64-year-old male patient in the setting of recent depression and paranoia. The patient was managed aggressively with multiple pressors in addition to both hydroxocobalamin and methylene blue, resulting in significant improvement of the patient’s hemodynamics. The results of our case presentation support that the use of methylene blue and hydroxocobalamin in conjunction with catecholamine vasopressors may be associated with a rapid increase in MAP and a reduction in the overall norepinephrine equivalent vasopressor requirements in severe refractory vasoplegia. Thus, this treatment approach could be an effective strategy for the management of patients with vasoplegic shock in the critical care setting. Future studies should aim to test the efficacy of hydroxocobalamin alone versus hydroxocobalamin and methylene blue, in conjunction with catecholamine vasopressors, to treat severe refractory vasoplegia.
